# Heat shock protein 90 promotes RNA helicase DDX5 accumulation and exacerbates hepatocellular carcinoma by inhibiting autophagy

**DOI:** 10.20892/j.issn.2095-3941.2020.0262

**Published:** 2021-08-15

**Authors:** Ting Zhang, Xinrui Yang, Wanping Xu, Jing Wang, Dawei Wu, Zhixian Hong, Shengxian Yuan, Zhen Zeng, Xiaodong Jia, Shanshan Lu, Rifaat Safadi, Sen Han, Zhihong Yang, Leonard M. Neckers, Suthat Liangpunsakul, Weiping Zhou, Yinying Lu

**Affiliations:** 1 Department of the Fifth Medical Center, General Hospital of PLA, Beijing 100039, China; 2 Division of Gastroenterology and Hepatology, Department of Medicine, Indiana University School of Medicine, Indianapolis 46202, USA; 3 Urologic Oncologic Branch, Center for Cancer Research, National Cancer Institute, Bethesda 20814, USA; 4 State Key Laboratory of Toxicology and Medical Countermeasures, Beijing Institute of Pharmacology and Toxicology, Beijing 100039, China; 5 The Third Department of Hepatic Surgery, Eastern Hepatobiliary Surgery Hospital, Shanghai 200438, China; 6 Hadassah Medical Organization, Hadassah Hebrew University Medical Center, Jerusalem 9112001, Israel; 7 Department of Biochemistry and Molecular Biology, Indiana University School of Medicine, Indianapolis 46202, USA; 8 Center for Synthetic and Systems Biology (CSSB), Tsinghua University, Beijing 100085, China

**Keywords:** Hepatocellular carcinoma, heat shock protein 90, RNA helicase DDX5, autophagy, β-catenin pathway

## Abstract

**Objective:**

Hepatocellular carcinoma (HCC), the main type of liver cancer, has a high morbidity and mortality, and a poor prognosis. RNA helicase DDX5, which acts as a transcriptional co-regulator, is overexpressed in most malignant tumors and promotes cancer cell growth. Heat shock protein 90 (HSP90) is an important molecular chaperone in the conformational maturation and stabilization of numerous proteins involved in cell growth or survival.

**Methods:**

DDX5 mRNA and protein expression in surgically resected HCC tissues from 24 Asian patients were detected by quantitative real-time PCR and Western blot, respectively. The interaction of DDX5-HSP90 was determined by molecular docking, immunoprecipitation, and laser scanning confocal microscopy. The autophagy signal was detected by Western blot. The cell functions and signaling pathways of DDX5 were determined in 2 HCC cell lines. Two different murine HCC xenograft models were used to determine the function of DDX5 and the therapeutic effect of an HSP90 inhibitor.

**Results:**

HSP90 interacted directly with DDX5 and inhibited DDX5 protein degradation in the AMPK/ULK1-regulated autophagy pathway. The subsequent accumulation of DDX5 protein induced the malignant phenotype of HCC by activating the β-catenin signaling pathway. The silencing of DDX5 or treatment with HSP90 inhibitor both blocked *in vivo* tumor growth in a murine HCC xenograft model. High levels of HSP90 and DDX5 protein were associated with poor prognoses.

**Conclusions:**

HSP90 interacted with DDX5 protein and subsequently protected DDX5 protein from AMPK/ULK1-regulated autophagic degradation. DDX5 and HSP90 are therefore potential therapeutic targets for HCC.

## Introduction

Liver cancer is one of the leading causes of cancer deaths in many countries, with the highest disease burden in East Asia. The most common type of primary liver cancer is hepatocellular carcinoma (HCC) comprising almost 90% of cases, followed by cholangiocarcinoma^1^. HCC occurs predominantly in patients with underlying chronic liver disease and cirrhosis, and the major causes of this disorder vary according to the geographic region^2,3^. In Asia, hepatitis B virus (HBV) is the major risk factor for HCC^2^, whereas hepatitis C virus, and nonalcoholic and alcoholic liver disease are the leading causes of HCC in Western countries^3^. The overall prognoses of patients with HCC depend on tumor staging and the underlying hepatic function^4^. While the overall survival of HCC patients has increased in the past decade, the overall prognosis remains poor^5^. Understanding the complex molecular mechanism of HCC pathogenesis is therefore of major importance, and may lead to future targeted therapies.

DEAD-box proteins are ubiquitous in RNA-mediated processes and function by coupling cycles of ATP binding and hydrolysis to changes in affinity for single-stranded RNA. They are the largest family of superfamily 2 helicases^6^. DEAD-Box Helicase 5 (DDX5) is a prototypic member of the DEAD-box family containing the Asp-Glu-Ala-Asp (D-E-A-D) helicase signature sequence^7^. It is ubiquitously expressed and plays multifunctional roles in several cellular processes^8^. As a component of RNA helicase, DDX5 acts as a transcriptional co-regulator with multiple transcription factors during the promotion of cancer cell proliferation^7,9,10^. DDX5 is frequently overexpressed in many cancers and contributes to disease pathogenesis and progression^11^.

The roles of DDX5 in the pathogenesis of HCC remain elusive. Its expression was shown to be associated with tumor size, tumor differentiation, and staging^12^. Despite extensive reports elucidating the role of DDX5 in promoting cancer progression, little is known about its function during HCC pathogenesis. At the time of this manuscript preparation, there was a report showing that DDX5 interacted with autophagic receptor p62 in promoting autophagy, independent of its RNA binding and helicase activity, while suppressing HCC tumorigenesis^13^. Additionally, DDX5 negatively correlated with p62/sequestosome 1 (SQSTM1) expression in HCC patients, and its expression was a biomarker in predicting prognosis after tumor resection^13^.

In the present study, we found that DDX5 protein was increased in HCC patients with high heat shock protein 90 (HSP90) expression, and was associated with higher tumor recurrence. We also report the novel post-translational regulation of DDX5 protein expression in HCC by an autophagic process, through its interaction with HSP90. DDX5 promotion of the malignant phenotype of HCC by activating the β-catenin signaling pathway and targeting DDX5 may therefore be used as a therapeutic strategy.

## Materials and methods

### Study cohort and human samples

A total of 24 surgical resection specimens of HCC and adjacent non-neoplastic liver tissues were obtained from patients who underwent surgical resection at the Fifth Medical Center, General Hospital of PLA (Beijing, China) and the Eastern Hepatobiliary Surgery Hospital (Shanghai, China) between 2009–2016. Baseline demographic and tumor characteristics of patients in our study are shown in **Table 1**. This study was approved by the Ethics Committee at each hospital (Approval No. 2016081D) and all patients provided informed consent prior to participation.

**Table 1:** Baseline demographic and clinical characteristics of the study cohort

Variables	
Age, years	49 ± 10.2
Gender	
Female	16.7%
Male	83.3%
BCLC stage	
A	37.5%
B	45.8%
C	16.7%
HBsAg positive	95.8%
Serum AFP, ng/dL	
< 10	29.1%
> 10	70.9%
Average tumor size, cm	5.4

### The Cancer Genome Atlas (TCGA) database analysis

Additional analyses from human samples were performed using TCGA. The RNASeq data from 371 HCC and 50 non-neoplastic tissues were analyzed.

### Plasmids, antibodies, and reagents

The pMXs-3Flag and pSUPER plasmids were kindly provided by Professor Hongjie Yao from the Chinese Academy of Sciences Key Laboratory of Regenerative Biology^14^. The detailed information regarding them is listed in **Supplementary Table S1**. MG132 (ubiquitin-proteasome inhibitor, working concentration: 10 µM), MRT68921 (unc-51-like kinase 1 inhibitor to block autophagy, working concentration: 1 µM), 5-amino-4-imidazolecarboxamide ribofuranoside (AICAR, autophagy inducer, working concentration: 100 µM), and STA9090 (heat shock protein 90, HSP90, inhibitor, working concentration: 10 µM) were purchased from Shanghai Selleck Chemicals (Shanghai, China).

### Characterization of protein-ligand docking

Characterization of protein-ligand docking between HSP90 and DDX5 was performed using ZDOCK software (http://zdock.umassmed.edu/software/)^15^. The B chain structure in the Protein Data Bank (PDB) archive crystal structure (2qf6) was chosen as the initial structure of HSP90, and the A chain structure in the PDB crystal structure (4a4d) was chosen as the initial structure of DDX5. After obtaining the optimized predictive complex configuration from the ZDOCK software, we next used the AMBER energy refinement program to identify the optimized structure of the HSP90-DDX5 complex.

### Laser scanning confocal microscopy (LSCM)

For cellular protein localization and expression, a laser scanning confocal microscope was used (TCS SP2/AOBS; Leica, Wetzlar, Germany) and images were collected and merged using Leica Application Suite X software.

### Xenograft tumor model

Male BALB/c-nu nude mice, 4 weeks of age, were purchased from Beijing Biotechnology (Beijing, China). The Huh7 and DDX5-deficient Huh7 hepatoma cell lines were cultured, washed, and resuspended at 1 × 10^7^ cells/mL in 0.9% sodium chloride. Aliquots of 0.1 mL (1 × 10^6^ cells/mouse) were subcutaneously injected with a 29-gauge needle into the flanks of 4~5-week-old mice. Tumor volumes (V) were calculated by caliper measurements of the longest (L) and the shortest (S) diameters of each tumor using the formula^16^: V = 0.5LS^2^. In some experiments, mice were injected with the STA9090 HSP90 inhibitor into the tail vein using a 30-gauge needle with a dosage of 10 mL/kg^17^. After treatment, tumor volumes were measured daily except for the weekend. All mice were sacrificed at day 20 of post-treatment. Animal experiments were approved by the Animal Committee of the Fifth Medical Center, General Hospital of PLA (Beijing, China) (Approval No. IACUC-2016-0014).

### Statistical analysis

All experiments were performed in triplicate unless otherwise stated. Statistical analysis was performed using SPSS statistical software for Windows, version 22.0 (SPSS, Chicago, IL, USA) and Prism 7 software (GraphPad, San Diego, CA, USA). Appropriate statistics such as the Mann-Whitney U test, Student’s *t*-test, and analysis of variance were used to determine the difference between/among groups for categorical and continuous variables. To determine the cumulative recurrence of HCC after resection, the Cox proportional hazards regression model was used. Data are presented as the mean ± standard error of the mean (SEM), unless otherwise indicated. A value of *P* < 0.05 was considered to be statistically significant. Other detailed methods are provided in the supplementary documents.

## Results

### DX5 mRNA and protein expressions in patients with HCC and its prognostic significance

**Figure 1:**
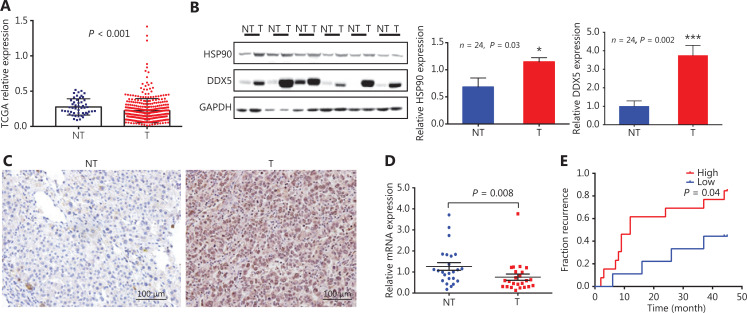
DDX5 mRNA and protein expression in patients with hepatocellular carcinoma (HCC) and its prognostic significance. (A) Relative expression of DDX5 mRNA in HCC using The Cancer Genome Atlas database from 371 HCC tissues compared to 50 non-neoplastic liver tissues; *P* < 0.001. (B) DDX5 and HSP90 protein expressions in HCC tissues (T) and surrounding non-neoplastic area (NT) detected by Western blot; *P < 0.05; ***P < 0.001. (C) Immunohistochemistry of DDX5 from HCC and non-neoplastic tissues (Scale bar: 100 μm). (D) The mRNA level of DDX5 in HCC surgical specimens detected by qRT-PCR (mean ± SEM, *n* = 24, *P* = 0.008). (E) The incidence of HCC recurrence after resection stratified by baseline DDX5 expressions in tumor tissues (cut-off value, T/NT= 1, *n* = 24, *P* = 0.04).

We first analyzed *DDX5* gene expressions using TCGA database from 371 HCC tissues, and compared them with 50 non-neoplastic liver tissues (**Figure 1A**). The relative expression of *DDX5* mRNA in HCC patients was significantly lower than that of the surrounding non-neoplastic areas (*P* < 0.001). Notably, we found that in tumor tissues from patients (*N* = 24) with higher expressions of HSP90, DDX5 expression was higher in tumor tissues (T) than in non-neoplastic liver tissues (NT) (**Figure 1B and C**; quantitative analysis, *P* = 0.002). In contrast, DDX5 mRNA expression in this cohort was also lower than tumor tissues (**Figure 1D**, *P* = 0.008). Furthermore, DDX5 protein expression was associated with a higher percentage of HCC recurrence after resection **(Figure 1E).**

### DDX5 interacts directly with HSP90

**Figure 2:**
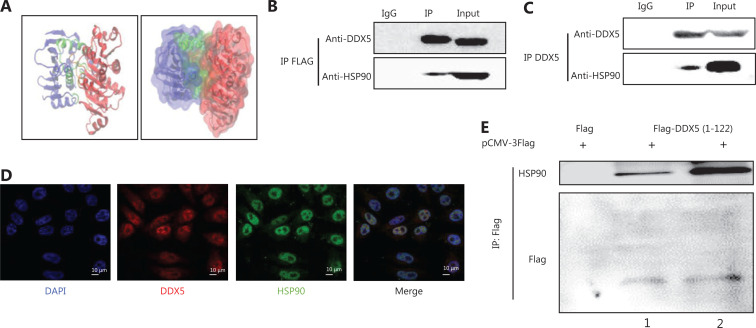
DDX5 interacts directly with HSP90. (A) Molecular docking between DDX5 and HSP90. The blue area represents HSP90, and the red area represents DDX5. The green and orange areas (arrows) represent the interaction between HSP90 and DDX5. (B and C) Co-immunoprecipitation between HSP90 with overexpressed DDX5-FLAG and endogenous DDX5. Lane IgG was a negative control; Lane IP was conducted using antibodies for FLAG (upper panel) or DDX5 (lower panel) antibodies; Lane input was the cell lysate (as a positive control). (D) Subcellular co-localization of DDX5 and HSP90 using confocal microscopy (Scale bar: 10 μm). HepG2 cells were stained with DAPI (blue, nuclear staining), DDX5 antibody (red), and HSP90 antibody (green). (E) A FLAG pull-down assay of truncated DDX5 (1-122 amino acids)-FLAG fusion fragment. Lane 2 is a double loading of lane 1.

Heat shock proteins, notably HSP90, are overly expressed in several tumor tissues^18–21^, so these chaperone proteins are thought to be a potential new target for cancer therapy^21,22^. Because of its function in maintaining the stability of other proteins, we next determined if HSP90 directly interacted with the DDX5 protein in HCC tissues, using ZDOCK software to determine potential protein-ligand docking (**Figure 2A**). We found direct interaction between these 2 proteins, with the interaction area involving a region of 57–67 amino acids (AAs). Protein complex immunoprecipitation experiments were then conducted to confirm the interaction between DDX5 and HSP90 (**Figure 2B and 2C**). An interaction was detected between both exogenous (DDX5-FLAG, **Figure 2B**) and endogenous DDX5 (**Figure 2C**) with HSP90. To determine the cellular protein localizations and expressions of DDX5 and HSP90, we conducted confocal microscopy of HepG2 cells (**Figure 2D**), and found that both proteins were primarily co-localized in the nucleus. As a final confirmatory step, we overexpressed the truncated DDX5-FLAG fusion fragment (1–122 AAs) in HepG2 cells. DDX5 protein was pulled down using anti-FLAG beads, and the cell lysates were immunoblotted using HSP90 and FLAG tag antibodies (**Figure 2E**). The results showed an interaction between HSP90 and the truncated DDX5-FLAG fusion protein, but not with FLAG tag alone. For the first time, our results showed the direct interaction between DDX5 and HSP90, suggesting that the HSP90 molecular chaperone may play a role in maturation or stabilization of the DDX5 protein, leading to an increase in its expression in HCC tissues.

### HSP90 inhibits the autophagic degradation of the DDX5 protein

**Figure 3:**
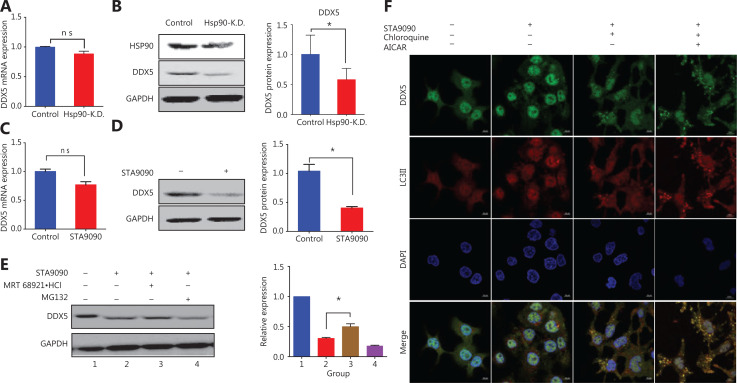
HSP90 inhibits the autophagic degradation of DDX5 protein. (A) The mRNA level of DDX5 after HSP90 knockdown with shRNA (Hsp90-K.D.), tested by qRT-PCR, using GAPDH as a reference gene. (B) The protein level of DDX5 after HSP90 knockdown (Hsp90-K.D.) (mean ± SD, *P = 0.0238). (C) The mRNA level of DDX5 after STA9090 treatment, tested by qRT-PCR, using *GAPDH* as a reference gene. (D) The protein level of DDX5 after STA9090 treatment (mean ± SD, *P = 0.0294). (E) The inhibitory effect of DDX5 expression in the presence of STA9090, an inhibitor of HSP90, was ameliorated in HepG2 cells treated with an inhibitor for autophagy, MRT68921, but not by MG132, an inhibitor of proteasomes. (F) Confocal microscopy of intracellular localization analysis of DDX5 and autophagosomes after treatment with STA9090, chloroquine, and AICAR (Scale bar: 10 μm). Chloroquine: inhibitor of lysosome; AICAR: agonist of autophagy. Arrow: DDX5 combined with autophagosomes.

Autophagy is a highly regulated intracellular pathway for protein degradation^23^. Several studies have reported that HSP90 plays an important role in protein homeostasis through autophagic machinery and the ubiquitin-proteasome pathway^24–26^. We thus hypothesized that DDX5 protein expression was regulated by HSP90. To confirm this possibility, we first used small interfering RNA (siRNA) for HSP90 to knockdown its expression in HepG2 cells (**Figure 3A and 3B**). The transfection efficiency was confirmed using Western blot analysis, which showed a significant decrease in HSP90 expression (**Figure 3B, right panel**). A deficiency in HSP90 protein did not interfere with *DDX5* mRNA levels (**Figure 3A**) but did lead to the reduction in DDX5 protein expression (**Figure 3B**). To confirm our finding, we used a pharmacological approach by treating HepG2 cells with STA9090, a specific HSP90 inhibitor, followed by determination of the mRNA and protein expressions of DDX5 (**Figure 3C and 3D**). Inhibition of HSP90 did not affect the levels of DDX5 transcripts, but did significantly hinder DDX5 protein expression, suggesting the important role of HSP90 in regulating DDX5 homeostasis.

To determine if HSP90 regulated the fate of the DDX5 protein, we treated HepG2 cells with specific inhibitors for proteasomes (MG132) and autophagy (MRT68921), the two major pathways of HSP90-regulated protein homeostasis in the presence and absence of STA9090 (**Figure 3E**). **Figure 3E** shows that DDX5 protein expression was significantly decreased in HepG2 cells treated with STA9090 (**Figure 3E, lane 2**). The expression of DDX5 was not altered in STA9090-treated HepG2 cells in the presence of the proteasome inhibitor, MG132 (**Figure 3E, lane 4**), when compared to STA9090-treated HepG2 cells alone. The inhibitory effect of DDX5 expression in the presence of STA9090 was ameliorated in HepG2 cells treated with an inhibitor for autophagy, MRT68921 (**Figure 3E, lane 3**). Taken together, our results suggested that HSP90 regulated DDX5 protein expression through an autophagic process rather than through a proteasomal pathway.

During autophagy, the cytosolic form of light chain 3 (LC3-I) is conjugated to phosphatidylethanolamine to form a LC3-phosphatidylethanolamine conjugate (LC3-II), which is recruited to autophagosomal membranes^27^. Autophagosomes fuse with lysosomes to form autolysosomes, and intra-autophagosomal components are degraded by lysosomal hydrolases when LC3-II in the autolysosomal lumen is degraded^27^. Thus, lysosomal turnover of the autophagosomal marker, LC3-II, reflects autophagic activity^27^. If the regulation of DDX5 protein expression by HSP90 is through an autophagic process, we reasoned that DDX5 should co-localize with autophagy markers such as LC3II. Cellular protein localization and expression of DDX5 and LC3II were therefore conducted using LSCM in the presence of chloroquine (inhibitor of fusion of autophagosomes with lysosomes) or AICAR (autophagy inducer) in STA9090-treated HepG2 cells (**Figure 3F**). We found that DDX5 in the cytoplasm was degraded by STA9090 treatment with few autophagosomes formed (arrow, **Figure 3F, second panel**). When HepG2 cells were treated with STA9090 and chloroquine, the significant increase in autophagosomes co-localized with DDX5 (arrows, **Figure 3F, third panel**). When we activated the autophagic process with AICAR, we found significantly decreased levels of DDX5 (**Figure 3F, fourth panel**).

### HSP90 inhibits DDX5 degradation and increases its expression through inhibition of the AMPK/ULK1-regulated autophagic pathway

**Figure 4:**
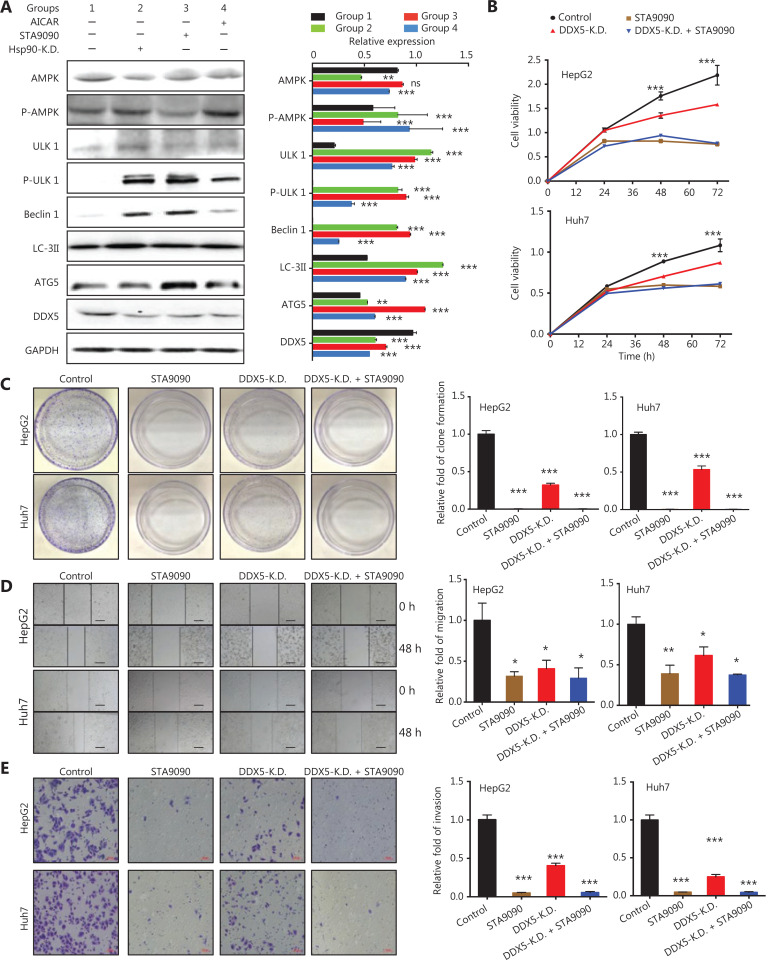
HSP90 inhibits DDX5 degradation and increases its expression by inhibition of the AMPK/ULK1-regulated autophagic pathway. (A) Western blot analysis of the protein expressions of AMPK, p-AMPK, ULK1, p-ULK1, Beclin1, LC3II, ATG5, and DDX5 in HepG2 cells treated with AICAR (positive control) STA9090 (a HSP90 inhibitor), and HSP90 knockdown (Hsp90-K.D). (B–E) Inhibition of DDX5 decreases cellular viability, migration, and invasion in the hepatoma cell line. (B) The cell viability of both HepG2 and Huh7 cell lines determined by the CCK8 assay. Circle, control group; square, STA9090 treatment group; upper triangle, DDX5 knockdown group (DDX5-K.D.); down triangle, the group treated with both DDX5-K.D. and STA9090. (C) Lack of DDX5 (DDX5-K.D. or STA9090 treatment) inhibited cellular proliferation in both HepG2 and Huh7 cells. (D) Cellular migration (D) and invasion (E) was inhibited in both Huh7 and HepG2 cells in DDX5 KD or STA9090 treatment (Scale bars, 200 μm). Data are from a representative experiment that was repeated 3 times with similar results (mean ± SD, *P < 0.05; **P < 0.01; ***P < 0.001).

HSP90 has previously been shown to interact with AMPK^28^, which then can regulate autophagy through direct phosphorylation of Unc-51-like autophagy activating kinase 1 (ULK1)^29^. We found that inhibition of HSP90 either with siRNA (HSP90 knockdown) or STA9090 in HepG2 cells increased the phosphorylation of AMPK and its downstream targets, ULK1, as well as markers for the autophagic pathway, Beclin1, LC3II, and ATG5 (**Figure 4A**). Importantly, DDX5 protein expression was reduced (**Figure 4A**). Together, our results suggested that HSP90 regulated DDX5 protein expression through the AMPK/ULK1 autophagic pathway.

### Inhibition of DDX5 decreases cellular viability, migration, and invasion in hepatoma cell lines

To determine the functions of DDX5, we next determined its effect on cellular viability in HCC cell lines. **Figure 4B** shows that inhibition of DDX5 using short hairpin RNA (shRNA) or pharmacological inhibition of the HSP90 inhibitor, STA9090 (**Figure 3D**), led to a significant decreases in cell viabilities in both HepG2 and Huh7 cells (**Figure 4B**). The cell viability in cells treated with shRNA (DDX5 KD) was approximately 30% and 21% lower than that of the controls in HepG2 and Huh7 cell lines, respectively (**Figure 4B**, *P* < 0.001 and 0.001, respectively, for HepG2 and Huh7 cells). STA9090-treated cells also had lower cell viability when compared to controls for both cell lines (*P* < 0.001 and 0.001, respectively, for HepG2 and Huh7 cells). Additionally, no cellular viability difference was observed in DDX5 KD cells treated with STA9090 compared to that of STA9090 treatment alone. Lack of DDX5 not only decreased the viability of hepatocellular cells, but it also inhibited cellular proliferation (**Figure 4C**). We also found that cellular migration and invasion were significantly inhibited in both Huh7 and HepG2 cells when DDX5 was inhibited (**Figures 4D and 4E).** Taken together, our results indicated the important role of DDX5 on cellular viability, migration, and invasion in hepatoma cells.

### DDX5 promotes the malignant phenotype of HCC by activating the β-catenin signaling pathway

Based on the clinical observation that DDX5 was highly expressed in HCC tissues, and associated with poor clinical outcomes as indicated by a higher HCC recurrence after resection (**Figures 1C–1E**). And, it was associated with cell viability, migration, and invasion (**Figures 4B–4E**). We therefore next conducted experiments to determine the molecular pathway associated with these processes.

**Figure 5:**
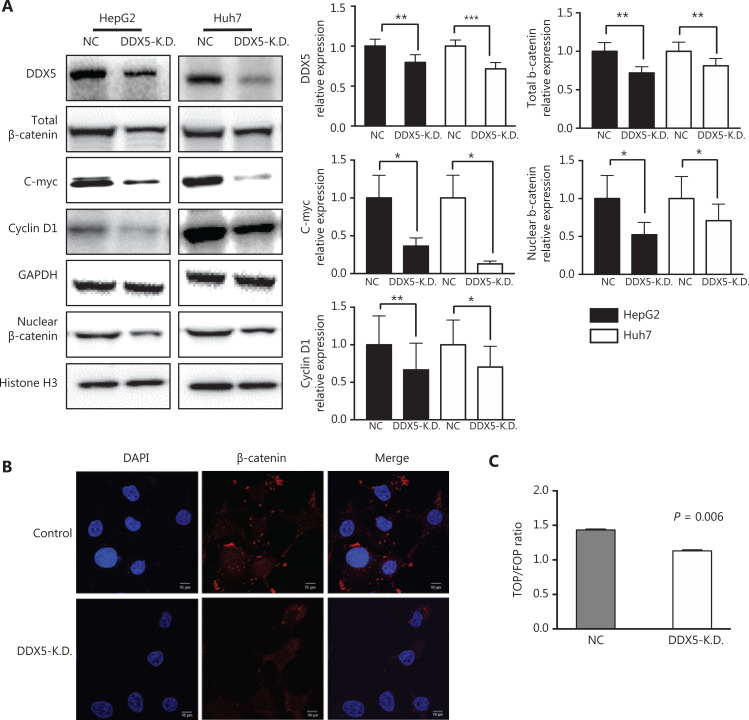
DDX5 promotes the malignant phenotype of hepatocellular carcinoma by activating the β-catenin signaling pathway. (A) Western blot analysis of DDX5 protein expressions in HepG2 and Huh7 cells, (total and nuclear) β-catenin, c-Myc, and cyclin D1 in groups of control, DDX5-K.D. and STA9090 treatments, respectively. GAPDH was used as an internal reference of total proteins, and Histone H3 was used as an internal reference of nuclear proteins; *P < 0.05; **P < 0.01; ***P < 0.001. (B) Immunofluorescence analysis of β-catenin expression in DDX5-K.D. (Scale bar: 10 μm). (C) DDX5-K.D. decreased the Top/Fop Flash activity (mean ± SD, *P* = 0.006).

Wnts are secreted signaling proteins, which are able to control cellular processes such as cell proliferation^30^. They act through a canonical, β-catenin signaling pathway. Wnt/β-catenin signaling also promotes cell migration and invasion^30,31^. We therefore determined whether the effect of DDX5 on cell viability, migration, and invasion was mediated through β-catenin signaling. We used the loss of function approach by knocking down DDX5 (DDX5-KD) using shRNA in HepG2 and Huh7 cells, and found that the expressions of β-catenin and its targets, c-Myc and cyclin D1, were significantly reduced in cells lacking DDX5, when compared with controls (**Figure 5A**). In the canonical Wnt cascade, β-catenin is the key effector responsible for transduction of the signal to the nucleus, where it triggers transcription of Wnt-specific genes^32^. We found that nuclear expression of β-catenin was significantly decreased in DDX5-KD in both cell lines, when compared with controls (**Figure 5A and 5B).** TOPFLASH and FOPFLASH vectors are a set of Tcf-reporter plasmids, and the TOPFLASH/FOPFLASH luciferase ratio has been widely used for the measurement of β-catenin signaling activity^33,34^. We found that the TOP/FOP luciferase ratio was significantly reduced in DDX5-KD HepG2 cells (*P* = 0.006; **Figure 5C**). Taken together, our results were consistent with the hypothesis that DDX5 promoted a malignant phenotype of HCC though activation of the β-catenin signaling pathway.

### Knockdown of DDX5 blocks *in vivo* tumor growth in a murine HCC xenograft model

**Figure 6:**
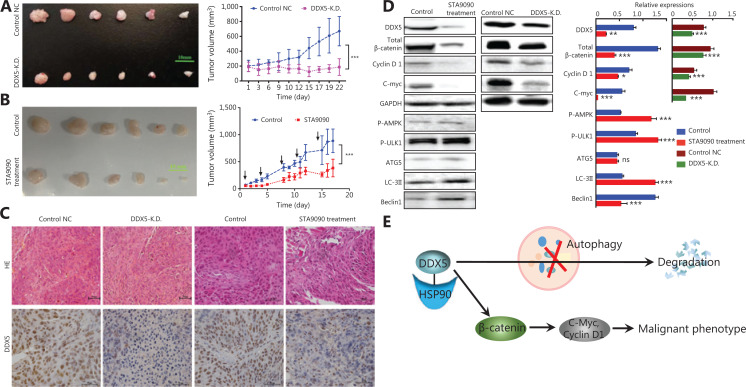
Knockdown of DDX5 blocks *in vivo* tumor growth in a murine hepatocellular carcinoma xenograft model. (A) DDX5-K.D. inhibited the tumor growth of the Huh7 mice xenograft model (left, photograph of tumors, scale bar: 10 mm; right, tumor volume, mean ± SD, ***P < 0.001). (B) STA9090 inhibited tumor growth in the Huh7 mice xenograft model (left, photograph of tumors, scale bar: 10 mm; right, tumor volume, mean ± SD, ***P < 0.001). (C) Immunohistochemical analysis of DDX5 in different groups of xenograft models (Scale bar: 50 μm). (D) Immunoblot analysis of DDX5 expression and the β-catenin signal pathway, and c-Myc and cyclin D1 expressions in STA9090-treated Huh7 cells and the DDX5-K.D. Huh7 xenograft mice models. Data are from a representative experiment that was repeated 3 times with similar results (mean ± SD, *P < 0.05; **P < 0.01; ***P < 0.001). (E) The schematic pathway summarizes the role of HSP90-DDX5 in promoting HCC. In HSP90^++^ HCC, the HSP90 inhibits DDX5 degradation and increases its expression through inhibition of autophagic pathway. DDX5 promotes the malignant phenotype of HCC by activating the β-catenin pathway.

To further investigate the function of DDX5 on tumor growth *in vivo*, we used a HCC xenograft model using Huh7 and Huh7 with DDX5-KD cell lines. Both cell lines were subcutaneously injected into the flank of nude mice. When the tumor nodes were visible approximately 24 h after injection, the average tumor volume was 147.2 ± 98.7 mm^3^ (in the Huh7 cell line) and 189.2 ± 64.6 mm^3^ (in the Huh7 cells with DDX5 knockdown). At the end of the experiments on day 21, the average tumor volume in the controls (Huh7 cells) was significantly higher than that in Huh7 cells with DDX5 knockdown (528.5 ± 172.5 mm^3^ *vs.* 203.9 ± 53.7 mm^3^, *P* < 0.001, **Figure 6A**).

To determine the effect of DDX5 inhibition with STA9090 on tumor growth, mice injected with Huh7 cells were treated with and without STA9090 using tail vein injections twice per week. The average tumor volume at baseline was 55.6 ± 37.1 mm^3^. At the end of the experiments, the average tumor volume in mice treated with STA9090 was significantly lower than that of the controls by approximately 46% (478.8 ± 342.2 mm^3^ *vs.* 887.0 ± 488.5 mm^3^, *P* < 0.001). Immunohistochemical staining for DDX5 in tumor tissues showed a significant reduction in DDX5 expressions in Huh7 with DDX5-KD and in mice treated with STA9090 (**Figure 6C**).

We also conducted experiments to determine β-catenin signaling in tumor tissues (**Figure 6D**). The expressions of β-catenin and its target, c-Myc and cyclin D1, in tumor tissues were significantly reduced in mice treated with STA9090 than those in the Huh7-DDX5-KD group. **Figure 6E** shows a schematic pathway summarizing the role of HSP90-DDX5 in promoting HCC.

## Discussion

In this study, we identified a novel role for the molecular chaperone, HSP90, in regulating DDX5 protein expression through an autophagic process in HCC patients. We also showed that DDX5 expression, through β-catenin signaling, was associated with cell migration and invasion, and was correlated with high HCC recurrence after hepatic resection.

DDX5 is a unique member of the highly conserved protein family, which is involved in many biological processes^7,12^. DDX5 also plays an important role in tumorigenesis, and it is overexpressed in various malignancies^7,35^. However, the exact role of DDX5 in the pathogenesis of HCC is controversial^12,13,35^. DDX5 is overexpressed at both transcriptional and translational levels in HCC tissues when compared with adjacent normal tissues^12^. Its expression has been correlated with tumor size, TNM staging, and tumor differentiation^12^. A high level of DDX5 expression is significantly correlated with poor overall survival, involving a mechanism related to the role of DDX5 in cell migration and invasion^12^. However, a recent study reported an opposite role of DDX5, and showed that DDX5 promoted autophagy and suppressed HCC tumorigenesis^13^. Using gain- and loss-of-function approaches, they reported that DDX5 overexpression was dramatically reduced, while DDX5 knockdown promoted cancer cell growth and tumorigenesis *in vitro* and *in vivo*^13^. In another study, HCCs from chronically HBV-infected patients showed a strong negative correlation between DDX5 mRNA levels, pluripotency gene expression, and liver tumor differentiation^35^. Furthermore, HBV patients with HCC expressing reduced DDX5 had poor prognoses after tumor resection^35^.

In the present study, we used TCGA database and a well characterized cohort of patients who underwent hepatic resection secondary to HCC, and showed that the relative expression of *DDX5* mRNA in HCC was significantly lower than that of the surrounding non-neoplastic areas. Our results were similar to a recent study showing a reduction of *DDX5* mRNA levels in HCC tissues, when compared with adjacent normal tissues^13^. However, when we determined DDX5 protein expression, to our surprise, we found that with high expression of HSP90, the expression of DDX5 protein in HCC tissues was increased, when determined using both Western blot and IHC techniques (**Figure 1B–1C and Supplementary Table S2**). One possible explanation of our results was that the degradation of DDX5 protein in HCC tissues was inhibited, when compared with adjacent non-HCC tissues.

To further characterize this mechanism, we showed an increase in the protein expression of HSP90, a chaperone protein, which plays an important role in protein degradation, in HCC and other cancers^24,36,37^. Notably, a study using dynamic proteomics to follow 100 proteins in human lung cancer cells following treatment with an HSP90 inhibitor showed that there was an increase in the level of DDX5 protein^38^. We therefore reasoned that the increase in HSP90 expression may have interfered with DDX5 protein degradation, and we found an interaction between HSP90 and DDX5 (**Figure 2**). Additionally, knocking down HSP90 led to changes in DDX5 protein expression without an alteration in the level of *DDX5* mRNA (**Figures 3A–3D**). It has been previously shown that HSP90 plays a role in autophagy *via* regulating the stability and activity of signalling proteins, and some HSP90 inhibitors can induce autophagy^25^. We also found that HSP90 regulated DDX5 protein expression through the inhibition of autophagy rather than the proteasome pathway. This mechanism is responsible for the increase in DDX5 protein levels in HCC tissues, which then promotes the malignant phenotype of HCC by activating the β-catenin signaling pathway. Importantly, the role of DDX5 in promoting cell proliferation and tumorigenesis by activating β-catenin does not seem to be specific to HCC, because it has been reported in other types of cancers^7^.

A key observation in our study on the prognostic role of DDX5 protein is that a low expression of DDX5 inhibited the malignant phenotype using *in vivo* tumor growth in the murine HCC xenograft model, leading to a good prognostic outcome after hepatic resection in patients with HCC. While our results are consistent with those reported by Xue et al.^12^, they contradict the results of others^13,35^. The results from these latter studies suggested that heterogeneity of the DDX5 protein in its function and mechanism was related to the pathogenesis of HCC, which depended on the patient population being studied. Given that the majority of our patients were those with chronic hepatitis B-induced HCC, similar to the other 2 reports^13,35^, it implied that the role of DDX5 was likely to be tumor- and patient-specific, rather than those of underlying liver diseases. This possibility was also suggested from studies determining the prognostic significance of protein NCK-associated protein 1 (NCKAP1) and outcomes in patients with HCC, which showed contradicting results, depending on the populations being studied^39,40^. Taken together, while modulation of DDX5 protein expression seems to be an attractive therapeutic strategy in patients with HCC, the approach in targeting DDX5 needs to be individualized, as its function in HCC pathogenesis varies with each patient.

## Conclusions

We report a novel mechanism of the inhibitory effect of HSP90 on DDX5 protein degradation resulting from the inhibition of autophagy. DDX5 promoted tumorigenesis, leading to poor outcomes with high HCC recurrence after hepatic resection. While our study suggested that blocking DDX5 protein expression may be beneficial in the treatment of HCC patients, the therapeutic strategy targeting DDX5 in patients with HCC needs to be individualized, because the functional role of this protein in HCC pathogenesis was unique and varied depending on the patient being treated.
