# Supplementary material for: Heat shock protein 90 promotes RNA helicase DDX5 accumulation and exacerbates hepatocellular carcinoma by inhibiting autophagy
Source: Cancer Biol Med. 2021 Aug 15;18(3):693–704. doi: 10.20892/j.issn.2095-3941.2020.0262 (PMC8330532; doi:10.20892/j.issn.2095-3941.2020.0262)
Supplement: Supplementary file 1 [file cbm-18-693-s001.pdf]

Supplementary materials

Methods

Cell culture

HepG2 and Huh7 Human HCC cells were purchased from the Type Culture Collection of the Chinese Academy of Sciences (Shanghai, China). Cells were cultured in DMEM medium (Hyclone, Logan, UT, USA) supplemented with 10% (v/v) fetal bovine serum, 100 μg/mL streptomycin, 100 U/mL penicillin, and 2 mm L-glutamine at 37 °C in a humidified incubator with 5% CO_2.

RNA isolation and quantitative RT-PCR (qRT-PCR) analysis

Total RNA was extracted using an AxyPrep total RNA Mini Extraction Kit (Axygen, Union City, CA, USA). The cDNA was obtained by reverse transcription of 1 μg total RNA using a RevertAid First Strand cDNA Synthesis Kit (Thermo Fisher Scientific, Waltham, MA, USA). The mRNA level of DDX5 was then detected by amplification and quantitation using a 7500 Real-Time PCR System (Applied Biosystems, Foster City, CA, USA). The relative quantitative gene expression was normalized to *GAPDH* as an internal control and calculated using the $2^{-\Delta\Delta Ct}$ method. The primer pairs used were as follows: DDX5 forward, 5′-3′: ATAACATAAAGCAAGTGAGCGACC; DDX5 reverse, 5′-3′: CCCTGGAACGACCTGAACCT; GAPDH forward, 5′-3′: TATGATGACATCAAGAAGGTGG; GAPDH reverse, 5′- 3′: CACCACCCTGTTGCTGTA.

Co-immunoprecipitation and immunoblotting analysis

The co-immunoprecipitation assay has been previously described (1). Briefly, the antigen-antibody complex was visualized using enhanced chemiluminescence Western blot detection reagents (GE Healthcare, Indianapolis, IN, USA). For immunoblotting of tissue lysates, 400 μL of RIPA Lysis Buffer (Solarbio, Beijing, China) mixed with protease and phosphatase inhibitors (TransGen Biotech, Beijing, China) was added to approximately 100–200 mm^3 of liver tissue. The protein concentration of the supernatant was determined using a BCA assay kit (Thermo Fisher Scientific).

Immunohistochemical analysis and immunofluorescence staining

Immunohistochemistry analysis was performed as previously described (2). Tissues were embedded in paraffin and cut into 5 μm sections. After deparaffinization, antigen retrieval was performed with sodium citrate buffer in a steamer for 20 min. Endogenous peroxidases were blocked by incubation for 30 min in 0.3% H_2O_2. After blocking, primary antibodies were incubated at 4 °C overnight, and then the slides were rinsed in wash buffer and incubated with secondary antibodies. Immunohistochemical labeling intensity was assessed by 2 experienced pathologists. Cells in 6-well culture slides were washed with 1× phosphate-buffered saline (PBS), fixed with 4% paraformaldehyde in PBS for 30 min, and then stored in PBS at 4 °C. Primary antibodies were incubated overnight at 4 °C and washed with 1× PBST, followed by addition of the secondary antibody. Images were acquired using a TCS SP2/AOBS microscope (Leica), using excitation wavelengths of 330 nm, 488 nm, and 515 nm.

Cell viability, colony formation, migration, and invasion assays

HCC cells seeded in 96-well plates with a density of 3,000 cells/well were cultured for 24, 48 and 72 h, then further incubated

Table S1 Antibody information

Specificity	Source	Company	CAT.
DDX5	Rabbit	Bethyl	A300-523A
Hsp90	Rabbit	CST	#4877
FLAG	Rabbit	CST	#14793
Hsp90	Mouse	Abcam	ab13492
AMPK	Mouse	Santa Cruz Biotechnology	Sc-74461
P-AMPK	Rabbit	Abcam	Ab133448
ULK1	Mouse	Santa Cruz	Sc-390904
P-ULK1	Rabbit	Abcam	Ab133747
Beclin1	Mouse	santa cruz	Sc-48341
LC-3II	Rabbit	Abcam	Ab192890
ATG5	Mouse	Santa Cruz	Sc-133158
C-myc	Rabbit	CST	#13987
Cyclin D1	Rabbit	CST	#2922
β-catenin	Rabbit	CST	#8480
Histone H3	Rabbit	proteintech	17168

Table S2 Immunohistochemical analysis of DDX5 expression in HCC tissues

IHC score	*n* (%) NT	*n* (%) T
0	8 (33.3)	0 (0)
1	8 (33.3)	2 (8.3)
2	5 (20.8)	4 (16.7)
3	3 (12.5)	8 (33.3)
4	0 (0)	10 (41.6)
Total	24	24

with 10 mg/mL CCK8 for 1 h (Dojindo, Nagasaki, Japan). The absorbance was measured at 450 nm using a Elx800™ spectrophotometer (Bioek, Winooski, VT, USA). For colony formation, the cells were seeded into 60 mm cell culture dishes (3,000 cells/dish) and cultured for 15 days. The cells were then fixed with 4% paraformaldehyde and stained with 1% Crystal Violet. The migration was assessed using a wound-healing assay. Images of the wound were recorded using a phase contrast microscope at different times (0 and 48 h). Wound closure/cell migration was evaluated using ImageJ software (National Institutes of Health, Bethesda, MD, USA), and cell invasion was evaluated using Matrigel Transwell assays as previously described (3). Briefly, cells (3×10^4) in serum-free DMEM were added to the upper chambers, which were coated with a thin layer of Matrigel matrix, and medium containing 10% FBS was added to the lower chambers. After 48 h of incubation (37 °C, 5% CO_2), the non-migrated cells were removed using 1× PBS. The upper chambers were fixed with 4% paraformaldehyde and stained with 1% Crystal Violet. Assays were repeated at least 3 times.

Gene knockdown by siRNA/shRNA

The *hsp90* gene was knocked down using small interfering RNA (siRNA) according to the manual supplied by Invitrogen (Carlsbad, CA, USA). The *ddx5* gene was knocked down using a short hairpin RNA (shRNA), and the plasmid for shRNA was synthesized by Sigma-Aldrich (St. Louis, MO, USA).

References

1. Bansal H, Bansal S, Rao M, Foley KP, Sang J, Proia DA, et al. Heat shock protein 90 regulates the expression of Wilms tumor 1 protein in myeloid leukemias. Blood. 2010; 116: 4591-9.
2. Lu Y, Xu W, Ji J, Feng D, Sourbier C, Yang Y, et al. Alternative splicing of the cell fate determinant Numb in hepatocellular carcinoma. Hepatology. 2015; 62: 1122-31.
3. Justus CR, Leffler N, Ruiz-Echevarria M, Yang LV. In vitro cell migration and invasion assays. J Vis Exp. 2014: 51046.
